# Designing Prefaculty Competencies for Diverse Learners Through a Modified Delphi Process

**DOI:** 10.1001/jamanetworkopen.2024.24003

**Published:** 2024-07-26

**Authors:** Rosa Lee, Raymond Lucas, Joel Dickerman, Lukejohn W. Day, Daniel Guzman, Pooja Kothari, LaTanya Love, William McDade, Ashley Rodgers, Monica Verduzco-Gutierrez, Lindy Zhang, John Paul Sánchez

**Affiliations:** 1 Columbia University Vagelos College of Physicians and Surgeons, New York, New York; 2 Department of Medicine, Columbia University Irving Medical Center, New York, New York; 3 George Washington University, Washington, DC; 4 St Mary Corwin and St Thomas More Hospitals, Pueblo, Colorado; 5 Zuckerberg San Francisco General Hospital and Trauma Center, California; 6 Division of Gastroenterology, University of California, San Francisco; 7 Columbia University Irving Medical Center, New York, New York; 8 Montefiore Medical Center, Bronx, New York; 9 McGovern Medical School at The University of Texas Health Science Center at Houston; 10 Accreditation Council for Graduate Medical Education, Chicago, Illinois; 11 Rush Medical College, Chicago, Illinois; 12 The Children’s Hospital at Montefiore, Bronx, New York; 13 University of Texas Health Science Center at San Antonio; 14 Sidney Kimmel Comprehensive Cancer Center and Department of Oncology, Johns Hopkins University School of Medicine, Baltimore, Maryland; 15 Universidad Central Del Caribe School of Medicine, Bayamón, Puerto Rico

## Abstract

**Importance:**

Faculty diversity in academic medicine may better prepare the next generation of equity-minded health care practitioners and leaders. Prefaculty development is an emerging concept to support trainees in achieving key knowledge, skills, and experiences to become successful faculty.

**Objective:**

To outline competencies, with corresponding milestones, to support the academic career development of learners, inclusive of racial, ethnic, sexual, and gender identities minoritized in medicine.

**Design, Setting, and Participants:**

Using a modified Delphi process, a national working group consisting of 13 members was established. The group used the published literature and listening sessions with diverse stakeholders to draft a set of competencies and milestones in July 2022. Diverse expert panelists reviewed the draft set over 2 rounds between September 2022 and January 2023. The group considered qualitative data to further refine the draft set between rounds. Consensus was reached when competencies and milestones were rated as agree or strongly agree on importance or appropriateness by 75% or greater of expert panelists after the second round. A final set of competencies and milestones was generated in February 2023. Data from round 1 were analyzed in October 2022 and data from round 2 were analyzed in January 2023.

**Main Outcomes and Measures:**

The development of prefaculty competencies with corresponding milestones by expert panel rankings and comments.

**Results:**

The national working group consisted of 13 members who represented diversity across racial, ethnic, and gender identities and academic and career tracks. The working group developed an initial set of 36 competencies and corresponding milestones across 12 domains. After 2 rounds, consensus among 46 expert panelists generated a final list of 32 competencies with corresponding milestones across 11 domains. A total of 26 panelists (56.5%) were women, 11 (23.9%) were Black or African American, 17 (37.0%) were Latina/o/x/e, Hispanic, or of Spanish origin, and 10 (21.7%) were White. Competency domains were divided into 2 groups: foundational (academic career choice and professional identity, mentorship, networking, financial skills, diversity and inclusion, personal effectiveness and self-efficacy, and leadership) and focused (education, community engagement, research, and clinical medicine). Consensus for inclusion or elimination of items was greater than 90% between the 2 rounds.

**Conclusions and Relevance:**

There was consensus among the working group and expert panelists regarding the importance and appropriateness of the competencies and milestones for diverse trainees to successfully obtain faculty positions. Institutions and national organizations can use these competencies as a framework to develop curricula that support diverse learners’ career development toward academia.

## Key Points

**Question:**

What competencies and milestones are needed for underrepresented minority trainees to succeed in achieving a career in academic medicine using a competency-based medical education framework?

**Findings:**

In this qualitative study, 46 expert panelists agreed on 32 competencies with corresponding milestones across 7 foundation domains (academic career choice and professional identity, mentorship, networking, financial skills, diversity and inclusion, personal effectiveness and self-efficacy, and leadership) and 4 focus area domains (education, community engagement, research, and clinical medicine).

**Meaning:**

This novel framework of competencies and milestones can be used to support diverse learners’ career development toward academic medicine.

## Teaser

This qualitative study evaluates competencies to support the academic career development of racial, ethnic, sexual, and gender identities minoritized in medicine.

## Introduction

Despite efforts to diversify the academic medicine workforce, the pathway from medical school through postgraduate training to faculty member is often unclear for trainees, with frequent deviations for trainees who identify as women, sexual and gender-minoritized individuals, and racial and ethnic-minoritized individuals who are underrepresented in medicine (UIM).^1^ Data from 2020 show that approximately one-third of the US population identified as a race other than White, yet this group composed under 18% of osteopathic and allopathic medical students and less than 10% of faculty.^2,3,4,5^ The Association of American Medical Colleges’ report, the State of Women in Academic Medicine, showed that while women made up 48% of medical school graduates, they composed only 41% of full-time US medical school faculty and only 18% of department chairs.^6^

Compared with their counterparts, UIM medical students have less intent to pursue academic careers upon matriculation and show a decline in intent thereafter.^7^ Reported reasons for this include limited awareness of academic medicine as a career option, perceived lack of competency to perform scholarly work or research, and lack of transparency in the faculty academic promotion process.^8^

Building the Next Generation of Academic Physicians, Inc (BNGAP) is a nonprofit organization with the mission to help diverse medical students and residents become aware of academic medicine career options and to provide them with resources to further explore and embark on an academic medicine career. In 2019, BNGAP leaders developed the conceptual framework of prefaculty development*,* defined as “providing learners with foundational self-efficacy, knowledge, skills, and experiences to be successfully appointed, and eventually promoted and tenured, within an academic institution.”^9,10^ In 2020 BNGAP formed the National Center for Pre-Faculty Development (NCPFD) to bring institutional leaders together to develop and implement best practices related to prefaculty development^11^ as a basis to rethink and improve efforts to prepare diverse learners to enter academic careers.

Competency-based medical education emphasizes learner outcomes, progression of ability, and learner-centeredness.^12^ While BNGAP and others have developed and published educational interventions to support trainees’ awareness of and skills toward academic medicine careers,^13,14,15^ a search by the authors yielded no set of published competencies to guide the development of prefaculty curricula. The purpose of our study was to design and validate, by consensus methodology, competencies and milestones consistent with success for UIM trainees who pursue academic careers using a competency-based medical education framework.

## Methods

We selected a modified Delphi process to develop and validate a set of prefaculty competencies and milestones in this qualitative study. While the modified Delphi process is not well defined,^16,17^ we describe our technique as one that establishes consensus for a set of competencies and milestones initially drafted by a workgroup and refined through several rounds of expert panel feedback. The modified Delphi technique was selected over other consensus methodologies because it can be done electronically, allows recruitment of a variety of experts across time and space, provides anonymity in the consensus process, and avoids groupthink and undue influence of more vocal members of an expert panel.^17^

To ensure rigor, we followed the guidance of van Zuuren and colleagues^18^ on procedures to increase the quality of reporting on consensus methodologies, and we used Standards for Quality Improvement Reporting Excellence (SQUIRE) reporting guidelines. This study was determined to be exempt from review by the University of New Mexico Office of Human Research. Participants were provided study information through an informed consent cover letter whereby continuing to the survey implied agreement to participate in the research study.

### Development of the National Working Group

A 13-member national working group (NWG) was formed in January 2022 to develop an initial draft of prefaculty competencies and milestones. Members were selected for expertise in relevant areas: competency-based medical education and curriculum development; diversity, equity, and inclusion; mentoring of diverse learners; leadership in osteopathic and allopathic medical schools; and the hiring of diverse faculty across tracks (eg, education, research, and clinical). NWG members included trainees and faculty leaders in undergraduate medical education, graduate medical education, research, and clinical environments. NWG members were purposefully selected to represent various racial, ethnic, and gender identities, different academic and career tracks, and various institutions (Table 1). Participants were asked to self-identify their race and ethnicity in the same question, “How do you identify?” Participants could respond with multiple options, which included: American Indian or Alaska native; Asian; Black or African American; Latina, Latino, Latinx, Hispanic or of Spanish origin; Middle Eastern or North African; Native Hawaiian or Other Pacific Islander; or White.

**Table 1.** Characteristics of National Working Group Members and Expert Panelists Involved With Designing Prefaculty Competencies and Milestones for Diverse Medical Students, Residents, and Fellows Through a 2-Round Modified Delphi Process, 2023

Characteristics	Participants, No. (%)		
	National working group members (n = 13)	Expert panelists	
		Round 1 (n = 58)	Round 2 (n = 46)
Racial or ethnic identity			
American Indian or Alaska Native	1 (7.7)	3 (5.2)	2 (4.3)
Asian	3 (23.1)	5 (8.69)	5 (10.9)
Black or African American	3 (23.1)	15 (25.9)	11 (23.9)
Latina/o/x/e, Hispanic, or of Spanish origin	4 (30.8)	18 (31.0)	17 (37.0)
Native Hawaiian or other Pacific Islander	0	1 (1.7)	1 (2.2)
White	3 (23.1)	16 (27.6)	10 (21.7)
Gender identity			
Man	7 (53.8)	23 (39.7)	18 (39.1)
Woman	6 (46.1)	33 (56.9)	26 (56.5)
Prefer not to answer	0	2 (3.4)	2 (4.3)
Sexual orientation			
Heterosexual	9 (69.2)	46 (79.3)	35 (76.0)
Gay, lesbian, bisexual, queer	4 (30.8)	7 (12.1)	6 (13.0)
Prefer not to answer	0	5 (8.6)	4 (8.7)
Region of current institution			
Central	1 (7.78)	10 (17.2)	7 (15.2)
Northeast	8 (61.5)	17 (29.3)	14 (30.4)
Southern	2 (15.4)	13 (22.4)	11 (23.9)
Western	2 (15.4)	18 (31.0)	14 (30.4)
Focus area of career within past 10 y^a^			
Administration	6 (46.1)	31 (53.4)	26 (56.5)
Community engagement	0	13 (22.4)	10 (21.7)
Diversity, equity, and inclusion	2 (15.4)	29 (50.0)	24 (52.1)
Health policy or advocacy	0	6 (10.3)	6 (13.0)
Research	2 (15.4)	21 (36.2)	13 (28.2)
Education	5 (38.5)	42 (72.4)	32 (69.6)
Clinical care	3 (23.1)	28 (48.3)	24 (52.1)
Current or recent (past 10 y) roles^a^			
Trainee (resident, fellow)	4 (30.8)	NA	NA
Clerkship director	0	4 (6.9)	4 (8.7)
Residency or fellowship director	2 (15.4)	5 (8.6)	6 (13.0)
Clinical service line director	0	4 (6.9)	2 (4.3)
Division director	0	4 (6.9)	4 (8.7)
Vice chair	1 (7.78)	6 (10.3)	5 (10.9)
Department chair	1 (7.7)	5 (8.6)	3 (6.5)
Assistant, associate, or senior associate dean	3 (23.1)	25 (43.1)	19 (41.3)
Medical school vice-dean or dean	2 (15.4)	4 (6.9)	2 (4.3)
Provost or vice president	1 (7.7)	4 (6.9)	1 (2.1)
Chief academic officer or chief medical officer	2 (15.4)	2 (3.4)	2 (4.3)

Abbreviation: NA, not applicable.

^a^ Questions allowed for respondents to select multiple options.

### Theoretical Framework

The NWG anchored its work in Lent’s^19^ social cognitive career theory (SCCT) which has been used in other studies investigating career choices of medical trainees^20,21,22^ and has influenced the initial concept of prefaculty development. SCCT proposes that one’s career choice is influenced by interactions with others and the environment through 3 linked variables: self-efficacy beliefs, outcome expectations, and goals.

### Literature Review

As a starting point, the project leaders introduced the concept of prefaculty development and provided the NWG group with peer-reviewed articles describing UIM learner perceptions of, and facilitators and barriers to, pursuing academic careers. They were also given the teaching tools listed in eAppendix 1 in Supplement 1 that had been previously developed by BNGAP to promote academic career choice among diverse learners. Each working group member was asked to review the provided materials, personally search recent literature related to career development in their academic focus (education; research; diversity, equity, and inclusion; clinical leadership; and so forth) and share these with the larger group to inform development of prefaculty competencies and milestones.

### Listening Sessions

Project leaders (R. Lee, R. Lucas, and J.S.) conducted listening sessions with diverse learners to consider their perspectives. These were conducted at the national conferences of the Latino Medical Student Association, Student National Medical Association, Association of American Indian Physicians, and BNGAP’s National LGBT Workforce Conference from March to May 2022. The sessions ranged in size from 8 to 15 students at each event. Listening sessions used the same 3 prompts for discussion: (1) What do you believe are the knowledge, skills, and attitudes that would help trainees like yourself successfully achieve a career in academic medicine? (2) Are there any specific knowledge, skills, or attitudes that you feel would be particularly important for students like you to acquire to succeed in academic medicine careers? (3) What do you perceive to be the greatest barriers to trainees like yourself in having a successful career in academic medicine? Formal qualitative analysis was not performed. However, notes from the discussions were compared with the reviewed literature^23,24,25,26,27,28^ and shared with the NWG.

### Generation of Initial Draft of Competencies and Milestones

Based on the literature review, stakeholder input, and NWG discussions, the NWG developed a list of general competency domains under which specific competencies could be categorized. Each NWG member, based upon interest and/or expertise, created draft competencies for 1 or more domains. These were presented to the full NWG, who through discussion and an iterative process created the initial set of competencies. The NWG then developed milestones for each competency at the beginner, intermediate, or advanced stage. Beginner milestones were best suited for a medical student or someone in the early stages of considering an academic career. Residents and fellows were considered in the intermediate stage. The advanced stage was identified as best suited for individuals who were applying for their first academic position or were in the early years of an existing position. Individuals could enter these stages at differing educational training levels depending on their prior exposure to the field of academic medicine or professional experiences and outcomes.

Domains were termed foundational if they were considered critical for any diverse learner entering a career in academic medicine regardless of their professional area of focus. Other domains were termed focus areas if they were considered important only to a specific faculty career track. The initial draft of competencies and milestones were completed in August 2022.

### Expert Panel

Experts were defined as faculty, deans, or administrators at US medical schools who had at least 5 years of experience mentoring or advising diverse learners plus expertise in 1 or more areas of medical education; student affairs; research; faculty affairs and faculty development; or diversity, equity, and inclusion. A pool of potential experts was identified through the professional networks of the NWG members, from the NCPFD center members, and from BNGAP’s listserv, which consists of faculty and senior administrators who have attended or presented at BNGAP’s Academic Career Development Seminars over the last 5 years. A recruitment invitation detailing the study, including the purpose of identifying competencies and milestones for diverse trainees, was emailed to potential experts. No incentives were given for participation in the expert panel.

### Delphi Process

There is no consensus in the literature on the optimal size of a Delphi expert panel,^16,17,18,29^ but given the large number of potential expert panelists balanced with a request to participate in at least 2 Delphi rounds with a large list of competencies, the NWG a priori established the optimal panel size to be 50 to 60 members. A draft of domains, competencies, and milestones was formatted for presentation, piloted with the BNGAP board of directors, and refined for clarity before distribution to the expert panel. The expert panelists were sent a list of proposed competency domains (each defined), individual competencies within each domain, and associated milestones for each competency with instructions including the purpose of the project. The beginner, intermediate, and advanced levels for the milestones were explained. Using a 5-point Likert scale, the expert panelists rated the importance of each competency domain and each competency (0: not applicable, cannot assess; 1: not important at all; 2: of low importance; 3: neutral; 4: important; 5: very important). Similarly, the expert panelists rated the appropriateness of milestones for each competency using a similar 5-point Likert scale. Expert panelists had an opportunity to provide free-text comments on each domain, competency, and milestone and were specifically asked to provide comments if they rated an item as anything less than important or appropriate. Expert panelists were also asked to suggest additional or alternative competencies and milestones. Expert panelists provided career and demographic data to ensure they met the definition of an expert.

The NWG defined consensus before the panel met. An item could be included as a competency or milestone if 75% or more of the expert panelists agreed or strongly agreed that the item was important or appropriate. Similarly, an item would be eliminated if 75% or more of the expert panelists disagreed or strongly disagreed that an item was important or appropriate. If an item did not achieve consensus for either inclusion or elimination, the NWG reviewed the experts’ comments to determine whether to eliminate, modify, revise, or keep an item in the subsequent rounds. Since attrition in participation between Delphi rounds could be expected,^17^ the NWG a priori established that a 75% or greater retention of expert panelists in subsequent rounds would be acceptable. At least 2 Delphi rounds were planned, with additional rounds planned only if less than 95% of the items achieved consensus for inclusion or elimination. The summary of ratings in the prior round was shared with the expert panelists when completing round 2 (or later if needed). Round 1 was completed in October 2022. Round 2 was completed in January 2023.

### Data Analysis

Study data were collected and managed using Research Electronic Data Capture (REDCap) a secure, web-based software platform designed to support secure data capture for research studies with automated export procedures for seamless data downloads to common statistical packages.^30,31^ Expert panel rankings and comments were exported from REDCap to Excel version 16.66.1 (Microsoft) for descriptive analysis. Data from round 1 were analyzed in October 2022 and data from round 2 were analyzed in January 2023.

## Results

An invitation email was sent to 529 potential experts, 62 (11.7%) of whom responded. The target panel size was met with 58 respondents (11.0% of total invited potential experts) who met the definition of an expert and rated all proposed competencies and milestones; 4 were eliminated for incomplete responses. A review of the 42 experts who shared their contact information demonstrated that 25 (59.5%) were from institutions outside of the NCPFD, suggesting a representative sample of experts beyond association with BNGAP. Respondents were asked to self-identify their race and ethnicity in a combined question with categories identical to those asked of the NWG members to determine if the expert panel represented diversity in racial and ethnic identities. Of the expert panelists who completed both rounds, 26 (56.5%) were women, 11 (23.9%) were Black or African American, 17 (37.0%) were Latina/o/x/e, Hispanic, or of Spanish origin, and 10 (21.7%) were White. Full characteristics of the expert panel are displayed in Table 1.

The NWG initially developed 12 competency domains consisting of 2 to 6 competencies per domain and corresponding milestones at the beginner, intermediate, and advanced level for each competency. Seven of these domains were foundational domains and 5 were focus-area domains.

Using preset thresholds described previously, in round 1 the experts achieved consensus (>75% agreed or strongly agreed) on the importance of all 7 foundational competency domains. They achieved consensus on 4 of the 5 focus-area domains. Health policy as a focus area and 2 competencies within that domain did not achieve the 75% consensus threshold. Based on this and the free-text comments, the NWG eliminated the focus competency domain of health policy and its corresponding competencies after round 1.

The remaining domains had a total of 33 competencies with corresponding milestones. Thirty-two competencies achieved consensus (with a range of 78% to 100% agreement for importance and 76% to 99% agreement for appropriateness of milestones). One competency under the domain of academic career choice, “Determines a ‘personal brand’ of importance to self and academic medicine,” was rated as important or very important by only 69% of experts, and its corresponding milestones were rated important or very important by 60%. Based on the free-text comments, some expert panelists took exception to the phrase “personal brand.” The NWG modified the competency by replacing that term with “authentic professional identity and vision” and resubmitted the competency in round 2. Although consensus for inclusion was achieved for all other competencies and milestones in round 1, some items were edited for round 2 based on free-text suggestions from the expert panelists to gain more clarity; to recategorize milestones to a different level in the beginner, intermediate, and advanced classification; and to reflect special considerations for minoritized learners.

Forty-six experts (79.3%) in round 1 completed round 2. In round 2, the 11 competency domains and 32 of 33 competencies and milestones achieved consensus for inclusion in the final set. The competency pertaining to “authentic professional identity and vision” again failed to achieve consensus by the experts. Based on the free-text comments, some experts felt it was too closely related to the competency “Assesses one’s own values and goals and their fit with an academic career” or that it was too advanced a competency for the prefaculty level. The NWG eliminated this competency. Having reached saturation with consensus for 97% of all items, the Delphi process ended after round 2 and resulted in a final set of 11 competency domains (Table 2) with 32 competencies. eTable 1, eTable 2, eTable 3, eTable 4, eTable 5, eTable 6, eTable 7, eTable 8, eTable 9, eTable 10, and eTable 11 in Supplement 1 list the final set of 32 foundational and focus-area competencies and their corresponding milestones with the level of agreement reached for each by the expert panel.

**Table 2.** Foundational and Focus-Area Competency Domains

Domain	Description
**Foundational competency**	
Academic career choice and professional identity	This domain addresses competencies and milestones related to knowledge of what is involved in an academic career, career options, and professional identity formation. It involves a process of self-reflection to determine professional goals and interests and how they may fit with an academic career. Professional identity encompasses the transformational journey whereby one integrates knowledge, skills, values, and behaviors to “think, feel, and act” like a member of the academic medicine community.
Mentoring	This domain addresses competencies that include knowledge and skills to successfully develop and engage in productive mentorship relationships that support entry and growth in academic medicine. These competencies include recognizing the value of mentorship in supporting professional growth for trainees, especially for those groups underrepresented in academic medicine.
Networking	This domain addresses competencies that include knowledge, skills, and experiences to build, grow, and modify networks that can aid in maximizing self-efficacy, opportunities, and promotion in academia.
Financial skills	This domain includes knowledge, skills, and experiences to help learners manage educational finances and debt; obtain a salary and starting package that is equitable and personally gratifying; and understand the finances associated with achieving mission-aligned actions and outcomes at an academic medicine center.
Diversity, equity, and inclusion	This domain includes understanding the importance of diversity, equity, and inclusion and justice in meeting the mission of academic medicine centers and planning an approach to engage in diversity, equity, and inclusion as a core component of one’s academic portfolio.
Personal effectiveness and self-efficacy	This domain includes personal effectiveness, which addresses knowledge, skills, and behaviors that support personal and professional growth, success, and well-being. This domain also includes self-efficacy, which involves assessing an individual’s belief in their capacity to execute behaviors necessary to produce specific academic career development performance attainments.
Leadership	This domain includes knowledge and skills that enable trainees to effectively engage in leadership activities to succeed in academia (see also personal effectiveness and self-efficacy).
**Focus-area competency**	
Education	This domain involves identifying and participating in educational activities (eg, teaching, curriculum development, administration) to clarify career choice and demonstrate accomplishments that will assist in securing an academic career as an educator. It involves developing educational scholarship and developing a teaching portfolio.
Community engagement	This domain involves identifying and participating in community engagement, community service, or advocacy activities to clarify career choice and demonstrate accomplishments in these areas that will assist in securing an academic career. It involves developing knowledge of different types of community engagement and how to effectively plan and evaluate community service or advocacy activities to eventually transform these into scholarship.
Research	This domain involves identifying and participating in research activities to clarify career choice and demonstrate accomplishments in research that will assist in securing an academic career. It involves developing knowledge and experience in key components of research, from identifying a research question to coauthoring a research paper.
Clinical medicine	This domain involves identifying and participating in clinical medicine activities that facilitate individuals’ recognition, skills, and experiences to succeed as a clinical faculty member. It involves developing knowledge and experience in key components of clinical medicine, from identifying a research question to transforming a health care model.

## Discussion

The modified Delphi process used in this qualitative study is an established consensus methodology often used in new and emerging areas for which empirical evidence does not yet exist. The ACCORD guidelines (Accurate Consensus Reporting Document) for reporting consensus methods in the biomedical literature^32^ were recently published, consisting of a checklist of items to “ensure clear and transparent reporting of consensus studies.” Although not available during the design and conduct phases of this study, we evaluated our methods and procedures using the ACCORD guidelines before publication and believe our modified Delphi procedure to be rigorous, clear, and transparent.

This is the first study to our knowledge to produce a set of competencies and milestones developed by consensus to support UIM learners in pursuing academic medicine careers along their journey from medical school through fellowship. These competencies were developed to assist diverse trainees to discern academic medicine career choices from nonacademic options, and to position trainees with a stronger foundation of self-efficacy, knowledge, skills, and experiences to embark on an academic career.

Many of these competencies and milestones could be useful for all learners to achieve an academic career, not just UIM learners. The authors considered this and believed a comprehensive set of competencies and milestones would be most useful in a competency-based medical education curriculum for prefaculty development for UIM learners, even if some components were more broadly applicable. We believe that the diversity of the listening session participants, working group membership, and expert panel ensured that the interests and needs of UIM learners were addressed and that the study aims were achieved. For example, within the broadly applicable competency domain of mentoring, there is a specific milestone, “compares and contrasts the benefits and challenges of identity-congruent and identity-noncongruent mentors.” Similarly, within the academic career choice and professional identity domain, one milestone is “reflects on the benefits and challenges of individuality and conformity for academic career success.”

Our study uniquely provides a set of competencies and milestones to guide an individual’s development from medical school through residency and/or fellowship to their first academic contract. The organization of milestones as beginner, intermediate, and advanced allows for individuals to apply the set of competencies and milestones based upon prior experiences irrespective of level of training. In addition, institutions can apply the framework in a longitudinal fashion and to support a small or large cohort of learners.

Our competencies and milestones align with a recent concept described by Tomlinson^33^ as “graduate capital,” defined as a model that identifies key lived experiences that confer benefits and advantages to graduates toward employability. The graduate capital model identifies domains of human capital, cultural capital, social capital, identity capital, and psychological capital. We were surprised to find strong alignment between our prefaculty competencies and these domains of graduate capital (Table 3). This alignment suggests that the acquisition of competencies in these prefaculty domains can confer capital to increase employability of UIM learners in academic medicine. Our leadership competency did not align with Tomlinson’s concept of graduate capital, but we believe it carries merit for health professionals given their role in driving clinical, educational, or research excellence.

**Table 3.** Comparison of Graduate Capital Model With Prefaculty Foundational Competency Framework^a^

Graduate capital model component	Prefaculty foundational competency domains
Human capital: includes hard skills of subject or occupation-specific knowledge and soft skills related to navigating one’s labor market, such as knowledge of how to apply for and access work opportunities	Academic career choice and professional identity Financial skills Education Clinical care Community engagement Research
Social capital: relationships and networks through which graduates can access employment opportunities	Mentoring Networking
Cultural capital: culturally valued knowledge that aligns the graduate to the workplace the graduate seeks to enter; behavioral qualities that signal likely social fit within their organization	Academic career choice and professional identity Diversity, equity, and inclusion
Identity capital: psychosocial aspects of graduate’s identity and extent to which graduate defines themselves in relation to their future employment	Academic career choice and professional identity Diversity, equity, and inclusion
Psychological capital: capacity for individuals to adapt to challenges and establish high locus of self-control and persistence; includes self-efficacy and resilience	Personal effectiveness and self-efficacy

^a^ Tomlinson M, McCafferty H, Fuge H, Wood K. Resources and readiness: the graduate capital perspective as a new approach to graduate employability. *J Natl Inst Career Educ Couns.* 2017;38(1):28-35. doi:10.20856/jnicec.3805

Competencies in the domains of academic career choice and professional identity, as well as diversity and inclusion, build identity capital, and this may be a particularly significant opportunity for UIM trainees. Identity capital may help UIM trainees consider their identity from an assets model, in which the diversity that they bring to an institution through their identities contributes to the excellence of an institution. This can potentially mitigate negative impacts such as the minority tax, which has been described as the burden of time and administrative work placed on minority faculty to advance diversity initiatives at their academic institutions on top of their usual academic responsibilities.^34,35^

### Limitations

This study has limitations. Our study design focused on representation in the NWG and expert panel by race and ethnicity, sex, sexual orientation, gender identity, and academic career focus, but we may have omitted other important facets of diversity in academic medicine that may have led to an incomplete listing of competencies or unintentional bias in competency development. Future study iterations might consider other important factors such as socioeconomic status, disability, and first-generation in medicine status.

Despite learner input through the listening sessions, our set of competencies and milestones was primarily informed by the published literature and validated by a set of senior faculty and administrators, which innately carries a time-based or generational bias. Fluidity in academic culture and climate, as exemplified through recent emphasis on well-being and recent anti–diversity, equity, and inclusion efforts, necessitates an openness to revising these competencies in a timely fashion for our current and future learners.

Once a competency met our preset threshold for consensus, it was included in the final list. The competencies were not weighted in terms of their importance. We did not attempt to determine if all or only a subset of competencies would be useful for all trainees and acknowledge there may be variability of importance of each competency for any individual trainee. This deserves further inquiry.

The competencies presented here focus on self-efficacy, knowledge, skills, and attitudes that may be acquired by individual trainees, and they do not emphasize important external factors that may be outside the locus of control for trainees. Institutional culture and climate are critical concurrent factors that are associated with UIM trainee progression into academia. We acknowledge the need for institutions to address and mitigate systemic factors within their own institutions that impede the entry and advancement of UIM into academic medicine careers.

## Conclusions

In this qualitative study using a modified Delphi method, we have developed a novel set of prefaculty competencies to promote the development of self-efficacy, knowledge, skills, and experiences that prepare UIM trainees to enter and succeed along academic medicine career tracks. We believe these competencies may serve as a roadmap for undergraduate and graduate medical education programs to develop a robust professional and academic career development curriculum for UIM trainees and may serve as a tool to empower trainees to identify their own areas for professional development and growth.

## Notes

Supplement 1. **eTable 1. **“Academic Career Choice and Personal Identity” Final Foundational Prefaculty Competencies and Milestones (Beginner, Intermediate, Advanced) Proposed by an Expert Panel, With Ratings by Round, From a Modified Delphi Process, 2023 **eTable 2. **“Mentoring” Final Foundational Prefaculty Competencies and Milestones (Beginner, Intermediate, Advanced) Proposed by an Expert Panel, With Ratings by Round, From a Modified Delphi Process, 2023 **eTable 3. **“Networking” Final Foundational Prefaculty Competencies and Milestones (Beginner, Intermediate, Advanced) Proposed by an Expert Panel, With Ratings by Round, From a Modified Delphi Process, 2023 **eTable 4. **“Financial Skills” Final Foundational Prefaculty Competencies and Milestones (Beginner, Intermediate, Advanced) Proposed by an Expert Panel, With Ratings by Round, From a Modified Delphi Process, 2023 **eTable 5. **“Diversity, Equity, and Inclusion” Final Foundational Prefaculty Competencies and Milestones (Beginner, Intermediate, Advanced) Proposed by an Expert Panel, With Ratings by Round, From a Modified Delphi Process, 2023 **eTable 6.** “Personal Effectiveness and Efficacy” Final Foundational Prefaculty Competencies and Milestones (Beginner, Intermediate, Advanced) Proposed by an Expert Panel, With Ratings by Round, From a Modified Delphi Process, 2023 **eTable 7. **“Leadership” Final Foundational Prefaculty Competencies and Milestones (Beginner, Intermediate, Advanced) Proposed by an Expert Panel, With Ratings by Round, From a Modified Delphi Process, 2023 **eTable 8. **“Education” Final Focus Area Prefaculty Competencies and Milestones Proposed by an Expert Panel, With Ratings by Round, From a Modified Delphi Process, 2023 **eTable 9. **“Community Engagement” Final Focus Area Prefaculty Competencies and Milestones Proposed by an Expert Panel, With Ratings by Round, From a Modified Delphi Process, 2023 **eTable 10.** “Research” Final Focus Area Prefaculty Competencies and Milestones Proposed by an Expert Panel, With Ratings by Round, From a Modified Delphi Process, 2023 **eTable 11. **“Clinical Medicine” Final Focus Area Prefaculty Competencies and Milestones Proposed by an Expert Panel, With Ratings by Round, From a Modified Delphi Process, 2023 **eAppendix. **BNGAP Publications

Supplement 2.
Data Sharing Statement
