# Supplementary material for: Designing Prefaculty Competencies for Diverse Learners Through a Modified Delphi Process
Source: JAMA Netw Open. 2024 Jul 26;7(7):e2424003. doi: 10.1001/jamanetworkopen.2024.24003 (PMC11282442; doi:10.1001/jamanetworkopen.2024.24003)
Supplement: Supplement 1. — eTable 1. “Academic Career Choice and Personal Identity” Final Foundational Prefaculty Competencies and Milestones (Beginner, Intermediate, Advanced) Proposed by an Expert Panel, With Ratings by Round, From a Modified Delphi Process, 2023 eTable 2. “Mentoring” Final Foundational Prefaculty Competencies and Milestones (Beginner, Intermediate, Advanced) Proposed by an Expert Panel, With Ratings by Round, From a Modified Delphi Process, 2023 eTable 3. “Networking” Final Foundational Prefaculty Competencies and Milestones (Beginner, Intermediate, Advanced) Proposed by an Expert Panel, With Ratings by Round, From a Modified Delphi Process, 2023 eTable 4. “Financial Skills” Final Foundational Prefaculty Competencies and Milestones (Beginner, Intermediate, Advanced) Proposed by an Expert Panel, With Ratings by Round, From a Modified Delphi Process, 2023 eTable 5. “Diversity, Equity, and Inclusion” Final Foundational Prefaculty Competencies and Milestones (Beginner, Intermediate, Advanced) Proposed by an Expert Panel, With Ratings by Round, From a Modified Delphi Process, 2023 eTable 6. “Personal Effectiveness and Efficacy” Final Foundational Prefaculty Competencies and Milestones (Beginner, Intermediate, Advanced) Proposed by an Expert Panel, With Ratings by Round, From a Modified Delphi Process, 2023 eTable 7. “Leadership” Final Foundational Prefaculty Competencies and Milestones (Beginner, Intermediate, Advanced) Proposed by an Expert Panel, With Ratings by Round, From a Modified Delphi Process, 2023 eTable 8. “Education” Final Focus Area Prefaculty Competencies and Milestones Proposed by an Expert Panel, With Ratings by Round, From a Modified Delphi Process, 2023 eTable 9. “Community Engagement” Final Focus Area Prefaculty Competencies and Milestones Proposed by an Expert Panel, With Ratings by Round, From a Modified Delphi Process, 2023 eTable 10. “Research” Final Focus Area Prefaculty Competencies and Milestones Proposed by an Expert Panel, With Ratings by Round, From [file jamanetwopen-e2424003-s001.pdf]

Supplemental Online Content

Lee R, Lucas R, Dickerman J, et al. Designing prefaculty competencies for diverse learners through a modified Delphi process. *JAMA Netw Open*. 2024;7(7):e2424003. doi:10.1001/jamanetworkopen.2024.24003

eTable 1. “Academic Career Choice and Personal Identity” Final Foundational Prefaculty Competencies and Milestones (Beginner, Intermediate, Advanced) Proposed by an Expert Panel, With Ratings by Round, From a Modified Delphi Process, 2023

eTable 2. “Mentoring” Final Foundational Prefaculty Competencies and Milestones (Beginner, Intermediate, Advanced) Proposed by an Expert Panel, With Ratings by Round, From a Modified Delphi Process, 2023

eTable 3. “Networking” Final Foundational Prefaculty Competencies and Milestones (Beginner, Intermediate, Advanced) Proposed by an Expert Panel, With Ratings by Round, From a Modified Delphi Process, 2023

eTable 4. “Financial Skills” Final Foundational Prefaculty Competencies and Milestones (Beginner, Intermediate, Advanced) Proposed by an Expert Panel, With Ratings by Round, From a Modified Delphi Process, 2023

eTable 5. “Diversity, Equity, and Inclusion” Final Foundational Prefaculty Competencies and Milestones (Beginner, Intermediate, Advanced) Proposed by an Expert Panel, With Ratings by Round, From a Modified Delphi Process, 2023

eTable 6. “Personal Effectiveness and Efficacy” Final Foundational Prefaculty Competencies and Milestones (Beginner, Intermediate, Advanced) Proposed by an Expert Panel, With Ratings by Round, From a Modified Delphi Process, 2023

eTable 7. “Leadership” Final Foundational Prefaculty Competencies and Milestones (Beginner, Intermediate, Advanced) Proposed by an Expert Panel, With Ratings by Round, From a Modified Delphi Process, 2023

eTable 8. “Education” Final Focus Area Prefaculty Competencies and Milestones Proposed by an Expert Panel, With Ratings by Round, From a Modified Delphi Process, 2023

eTable 9. “Community Engagement” Final Focus Area Prefaculty Competencies and Milestones Proposed by an Expert Panel, With Ratings by Round, From a Modified Delphi Process, 2023

eTable 10. “Research” Final Focus Area Prefaculty Competencies and Milestones Proposed by an Expert Panel, With Ratings by Round, From a Modified Delphi Process, 2023

eTable 11. “Clinical Medicine” Final Focus Area Prefaculty Competencies and Milestones Proposed by an Expert Panel, With Ratings by Round, From a Modified Delphi Process, 2023

eAppendix. BNGAP Publications

© 2024 Lee R et al. *JAMA Network Open.*

This supplemental material has been provided by the authors to give readers additional information about their work.

© 2024 Lee R et al. *JAMA Network Open.*

eTable 1. "Academic Career Choice and Personal Identity" Final Foundational Prefaculty Competencies and Milestones (Beginner, Intermediate, Advanced) Proposed by an Expert Panel, With Ratings by Round, From a Modified Delphi Process, 2023*

Competency	Beginner	Intermediate	Advanced	Delphi Ratings
Assesses one's own values and goals and their fit with an academic career	● Describes several academic career roles and their typical responsibilities (e.g., academic focuses, leadership roles, etc.)<br>● Identifies and engages in activities that build foundational knowledge and skills for an academic career choice<br>● Assesses one's own values and goals and their fulfillment through an academic medicine career	● Evaluates gaps in knowledge to pursue desired academic career focus<br>● Assesses gaps in knowledge, skills, and experiences to make a more informed decision about a particular career focus<br>● Reflects on whether academic-related activities fit with one's own values and goals	● Evaluates and chooses an appropriate academic focus for first academic position aligned with personal values and career aspirations	**Round 1**<br>Competency: 93%<br>Milestones: 90%<br>**Round 2**<br>Competency: 98%<br>Milestones: 94%
Navigates the integration of personal and professional Identity and academic medicine culture	● Reflects on differences and similarities between the culture of academic medicine/academic medical centers and one's own lived experiences and personal and professional identity<br>● Reflects on the benefits and challenges of individuality and conformity for academic career success	● Develops strategies to affirm one's personal and professional identity within academic medicine culture	● Optimizes value-based alignment of personal interests and values with the institutional mission and goals at a chosen academic medical center and/or its community<br>● Identifies ways to improve the climate of a chosen academic medical center through involvement in professional activities	**Round 1**<br>Competency: 88%<br>Milestones: 99%<br>**Round 2**<br>Competency: 87%<br>Milestones: 89%
**Competency**	**Beginner**	**Intermediate**	**Expert**	**Delphi Ratings**
Develops an action plan for securing an academic position that aligns with one's values	● Explores different timelines, applications, and action plans towards reaching an academic position in a chosen specialty<br>● Identifies role models, advisors, mentors, and/or sponsors in academic career positions that are of personal and professional interest (see also competencies on mentoring)	● Prioritizes potential academic positions in target specialty and geographic area<br>● Identifies network of support at academic medicine centers of particular interest<br>● Identifies 3 to 4 members of the academic medicine community who will write outstanding recommendation letters<br>● Drafts a personalized timeline and action plan for the period of medical	● Submits completed, competitive applications for desired academic positions<br>● Tailors application, interview, and other communication correspondences to optimize value-based alignment between self and the intended institution for an academic position<br>● Analyzes and ranks offer packages based on equity (e.g., pay,	**Round 1**<br>Competency: 97%<br>Milestones: 99%<br>**Round 2**<br>Competency: 94%<br>Milestones: 96%

© 2024 Lee R et al. *JAMA Network Open.*

	● Maintains updated documentation to support components for an application for their first academic medicine position (e.g., mission statement, CV, teaching/ research portfolio, etc.)	school, residency, and/or fellowship training towards securing an academic position<br>● Evaluates the climate of potential workplaces and how it aligns with one's values	resources, titles) and value-based alignment<br>● Drafts 5-year academic career plan	

* There were 58 respondents in round 1 and 46 respondents in round 2 of the Delphi process. Delphi ratings are expressed as percentage that agree or strongly agree with the competency or milestone.

© 2024 Lee R et al. *JAMA Network Open.*

eTable 2. “Mentoring” Final Foundational Prefaculty Competencies and Milestones (Beginner, Intermediate, Advanced) Proposed by an Expert Panel, With Ratings by Round, From a Modified Delphi Process, 2023*

Competency	Beginner	Intermediate	Advanced	Delphi Ratings
Demonstrates knowledge of different mentoring relationships to support a career in academic medicine	● Describes the role of mentors in developing careers in academic medicine, particularly for diverse learners<br>● Explains the roles and expectations for both mentor and mentee<br>● Describes facilitators and barriers to effective mentor-mentee relationships<br>● Compares and contrasts the benefits and challenges of identity-congruent and noncongruent mentors	● Differentiates mentors from advisors, coaches, and sponsors<br>● Applies different mentoring relationships to individual needs, including individual-, group-, peer-mentoring for professional development	● Evaluates the type of mentoring needed to succeed in first academic position	**Round 1**<br>Competency: 95%<br>Milestones: 91%<br>**Round 2**<br>Competency: 96%<br>Milestones: 94%
Engages in mentorship activities that will support a future academic career	● Identifies individuals who may serve as mentors or mentees<br>● Initiates and engages in relationship with a mentor to establish and achieve specific goals relating to personal and/or professional development	● Seeks and utilizes feedback to improve mentorship relationship<br>● Demonstrates effective communication skills to develop, maintain, and enhance a mentorship relationship<br>● Establishes and nurtures a mentorship network<br>● Uses different mentors and mentoring types depending on personal, professional, academic, research, or other needs	● Evaluates the stage of a mentorship relationship (initiation, negotiation, growth, closure) with mentors<br>● Knows and applies different strategies to move between stages of a mentoring relationship	**Round 1**<br>Competency: 100%<br>Milestones: 90%<br>**Round 2**<br>Competency: 98%<br>Milestones: 96%

* There were 58 respondents in round 1 and 46 respondents in round 2 of the Delphi process. Delphi ratings are expressed as percentage that agree or strongly agree with the competency or milestone.

© 2024 Lee R et al. *JAMA Network Open.*

eTable 3. "Networking" Final Foundational Prefaculty Competencies and Milestones (Beginner, Intermediate, Advanced) Proposed by an Expert Panel, With Ratings by Round, From a Modified Delphi Process, 2023*

Competency	Beginner	Intermediate	Advanced	Delphi Ratings
Recognizes the value of different networks in developing a successful academic career	• Describes the value of networking to increase visibility, opportunities, and productivity<br>• Recognizes tools and resources to build and grow professional networks<br>• Compares and contrasts the value of institutional, local, regional, and national networks<br>• Compares and contrasts the value of different networks by specialty and identity to ensure personal and professional fulfillment in academic medicine	• Appraises existing networks to better understand gaps in support to maximize academic productivity<br>• Prioritizes different networks depending on current academic career interests, goals, or actions	• Evaluates existing networks, including professional social networks, to determine which can support the early phase of a first academic position	**Round 1**<br>Competency: 90%<br>Milestones: 84%<br>**Round 2**<br>Competency: 96%<br>Milestones: 93%
Identifies and engages in networking activities to maximize self-efficacy, academic opportunities, and promotion	• Engages in networking across different communities (including identity and specialty; congruent and noncongruent networks) to expand one's professional support and outcomes	• Chooses social media platforms to expand professional network<br>• Analyzes how networks' support and associated activities influence one's own self-efficacy in pursuing an academic career<br>• Modifies networks depending on alternate goals, actions, and outcomes of interest	• Utilizes institutional, local, or regional networks to achieve relevant goals, actions, and outcomes<br>• Establishes a network to support success in scholarship activities expected in an academic career	**Round 1**<br>Competency: 88%<br>Milestones: 76%<br>**Round 2**<br>Competency: 89%<br>Milestones: 85%

* There were 58 respondents in round 1 and 46 respondents in round 2 of the Delphi process. Delphi ratings are expressed as percentage that agree or strongly agree with the competency or milestone.

© 2024 Lee R et al. *JAMA Network Open.*

eTable 4. "Financial Skills" Final Foundational Prefaculty Competencies and Milestones (Beginner, Intermediate, Advanced) Proposed by an Expert Panel, With Ratings by Round, From a Modified Delphi Process, 2023*

Competency	Beginner	Intermediate	Advanced	Delphi Ratings
Effectively manages finances during training to facilitate a viable and gratifying early academic career	● Constructs a budget to analyze one's own income and expenses<br>● Recognizes the influence of educational debt on career choice<br>● Lists financial aid options and available scholarships<br>● Recognizes terms and conditions of medical education loans, loan repayment, loan consolidation, and loan forgiveness programs	● Assesses expenses in a way that balances debt burden, well-being, and capacity for engaging in activities that help build success<br>● Describes financial compensation for primary care versus specialty care, and academic practice versus private practice<br>● Analyzes the finances and salaries for various academic tracks and leadership positions to determine potential options	● Constructs a financial plan for the first 5 years of academic practice that provides a gratifying early career	**Round 1**<br>Competency: 84%<br>Milestones: 83%<br>**Round 2**<br>Competency: 83%<br>Milestones: 83%
Successfully negotiates equitable starting salary and package for first academic position	● Lists negotiable and nonnegotiable components of an academic package<br>● Recognizes how academic faculty/ leaders are financially compensated based on role, responsibilities, productivity, and other factors<br>● States timeline and process for negotiating and completing final package	● Identifies and prepares for barriers to achieving an equitable salary and package<br>● Constructs a budget of potential income sources (e.g., consulting opportunities, royalties, speaking engagements, etc.) and expenses associated with being in academic practice	● Creates and benchmarks preferred salary and package for first academic position<br>● Successfully negotiates a starting salary and package for an academic position<br>● Evaluates life and disability insurance and other common benefits<br>● Judges whether starting salary and package is equitable and comparable to external benchmarks, based on one's identity and achievement	**Round 1**<br>Competency: 88%<br>Milestones: 78%<br>**Round 2**<br>Competency: 89%<br>Milestones: 85%
**Competency**	**Beginner**	**Intermediate**	**Advanced**	**Delphi Ratings**
Demonstrates and applies knowledge of academic health center finances and how they can be leveraged	● Recognizes the difference between pay for performance and value-based care in healthcare	Illustrates how activities of interest can be financially supported through a department or office	● Formulates a plan to gain financial support for academic-related goals, actions, or	**Round 1**<br>Competency: 84%<br>Milestones: 78%<br>**Round 2**

© 2024 Lee R et al. *JAMA Network Open.*

to support an academic medicine career	• Describes finances of an academic department / medical school office	• Describes how to document for optimal medical reimbursement	outcomes to be achieved through a department or office<br>• Is aware of different entities that contribute salary support to an academic position	Competency: 89%<br>Milestones: 80%

* There were 58 respondents in round 1 and 46 respondents in round 2 of the Delphi process. Delphi ratings are expressed as percentage that agree or strongly agree with the competency or milestone.

© 2024 Lee R et al. *JAMA Network Open.*

eTable 5. "Diversity, Equity, and Inclusion" Final Foundational Prefaculty Competencies and Milestones (Beginner, Intermediate, Advanced) Proposed by an Expert Panel, With Ratings by Round, From a Modified Delphi Process, 2023*

Competency	Beginner	Intermediate	Advanced	Delphi Ratings
Demonstrates knowledge of why DEI is important to healthcare and academic medicine	• Recognizes the current state, benefits, and challenges of diversity and inclusion in the academic healthcare workforce<br>• Explains how a diverse workforce can aid an academic medical center in achieving mission, values, accreditation standards, and excellence	• Illustrates through verbal or written communication how one's lived experiences and identity can support the academic medicine center and surrounding community<br>• Assesses how to be an engaged diverse academic medicine community member while mitigating imposter syndrome, tokenism, minority taxation, etc.	• Evaluates a potential employing academic medical center's goals, actions, and outcomes in achieving a diverse, inclusive, and equitable workforce	**Round 1**<br>Competency: 93%<br>Milestones: 97%<br>**Round 2**<br>Competency: 94%<br>Milestones: 96%
Explores how DEI as a topic can integrate in an academic career	• Describes the titles, responsibilities, activities, and deliverables of faculty/leaders engaged in DEI-related work	• Engages in DEI activities and scholarly work on the institutional, local, or regional level	• Formulates a plan to engage in DEI-related work as a part of their academic portfolio and that is supported through their starting package	**Round 1**<br>Competency: 78%<br>Milestones: 91%<br>**Round 2**<br>Competency: 78%<br>Milestones: 85%

* There were 58 respondents in round 1 and 46 respondents in round 2 of the Delphi process. Delphi ratings are expressed as percentage that agree or strongly agree with the competency or milestone.

© 2024 Lee R et al. *JAMA Network Open.*

eTable 6. "Personal Effectiveness and Efficacy" Final Foundational Prefaculty Competencies and Milestones (Beginner, Intermediate, Advanced) Proposed by an Expert Panel, With Ratings by Round, From a Modified Delphi Process, 2023*

Competency	Beginner	Intermediate	Advanced	Delphi Ratings
Demonstrates goal-directed behaviors	● Creates specific, measurable and actionable goals that align with securing a future academic career<br>● Enacts strategies to achieve goals<br>● Effectively monitors progress towards goal achievement	● Updates goals as academic career interests change or evolve<br>● Reflects on ability to achieve prior goals and adjusts behaviors towards increased goal attainment	● Revises goals from focus on securing an academic position towards those aligned with early career success	**Round 1**<br>Competency: 97%<br>Milestones: 93%<br>**Round 2**<br>Competency: 91%<br>Milestones: 89%
Practices effective time and attention management	● Routinely engages in short- and long-term time and task planning<br>● Effectively uses time management tools and strategies to reliably complete one's duties<br>● Identifies and avoids distractions and procrastination that interfere with goal achievement	● Manages time and attention in a manner that maximizes both professional goal achievement and optimal personal wellness<br>● Appropriately revises time and task planning when external demands are high or unexpected things occur	● Reframes time and attention to align with specific career track and career expectations<br>● Plans timeline for first 5 years of academic position	**Round 1**<br>Competency: 93%<br>Milestones: 90%<br>**Round 2**<br>Competency: 96%<br>Milestones: 91%
Demonstrates effective oral and written communication skills	● Speaks and writes in a clear and concise fashion<br>● Recognizes the importance of tailoring communication to audience<br>● Compares and contrasts effective and ineffective communication techniques	● Identifies when communication is ineffective and adjusts content or communication style as needed<br>● Uses various modalities—stories, emotions, persuasion, and evidence—appropriately and effectively in oral and written communications<br>● Demonstrates culturally competent and effective communication with multiple audiences	● Effectively uses oral and written communications with different communities by specialty or identity<br>● Effectively uses oral and written communications with different communities on the local, regional, and/or national level<br>● Communicates with the media in accordance with institutional guidelines	**Round 1**<br>Competency: 97%<br>Milestones: 91%<br>**Round 2**<br>Competency: 96%<br>Milestones: 96%
Demonstrates emotional intelligence	● Is self-aware of one's own emotions and how they may impact their decisions and interactions with others<br>● Describes emotional intelligence and its role in personal effectiveness	● Adjusts body language and spoken or written communications appropriately in difficult situations<br>● Seeks feedback and makes improvements after challenging	● Applies emotional intelligence to direct a team in completing academic-related projects	**Round 1**<br>Competency: 95%<br>Milestones: 86%<br>**Round 2**<br>Competency: 91%<br>Milestones: 85%

© 2024 Lee R et al. *JAMA Network Open.*

Competency	Beginner	Intermediate	Advanced	Delphi Ratings
		communications or stressful situations<br>● Recognizes the emotional state and nonverbal cues from others as a basis towards increased empathy		
Utilizes feedback for professional growth	● Recognizes the importance of feedback for academic career development<br>● Develops strategies to seek feedback regularly<br>● Maintains an open mind for positive and negative feedback	● Reflects on when and where feedback is needed depending on stage of academic career development<br>● Uses negative feedback as an opportunity for growth rather than a source of self-doubt	● Applies feedback to enhance academic career success	**Round 1**<br>Competency: 95%<br>Milestones: 84%<br>**Round 2**<br>Competency: 98%<br>Milestones: 87%
Develops self-efficacy	● Identifies prior personal experiences/ tasks that align with an academic medicine career and why these prior experiences/tasks were successful<br>● Identifies a person achieving an academic career similar to that which one hopes to achieve<br>● Reflects on both positive and negative emotions that may impact academic career progression (e.g., fear, anxiety, imposter syndrome, self-talk)	● Recognizes how certain experiences/ traits will enable one to be successful in current or future academic career-related tasks<br>● Identifies resources and tools to address negative traits that may hinder academic career progression	● Seeks out and participates in academic medicine activities that promote growth and manages any potential negative emotions that are associated with it	**Round 1**<br>Competency: 91%<br>Milestones: 86%<br>**Round 2**<br>Competency: 87%<br>Milestones: 85%

* There were 58 respondents in round 1 and 46 respondents in round 2 of the Delphi process. Delphi ratings are expressed as percentage that agree or strongly agree with the competency or milestone.

© 2024 Lee R et al. *JAMA Network Open.*

eTable 7. “Leadership” Final Foundational Prefaculty Competencies and Milestones (Beginner, Intermediate, Advanced) Proposed by an Expert Panel, With Ratings by Round, From a Modified Delphi Process, 2023*

Competency	Beginner	Intermediate	Advanced	Delphi Ratings
Develops personal leadership style	• Identifies different styles of leadership<br>• Identifies one’s leadership style, including strengths and weaknesses<br>• Recognizes opportunities for leadership at one’s institution<br>• Joins group/activity with leadership opportunities	• Obtains leadership position in a self-driven initiative or a group / organization with a clearly defined role<br>• Modifies personal leadership style to meet the needs of a project or achieve a different outcome<br>• Utilizes leadership position to support and develop growth of other individuals or an organization	• Seeks feedback on how their leadership style may compromise or support their success in academia	**Round 1**<br>Competency: 88%<br>Milestones: 86%<br>**Round 2**<br>Competency: 89%<br>Milestones: 89%
Manages teams effectively	• Works collaboratively with others on a team<br>• Provides constructive feedback to others on a team	• Promotes critical thinking, questioning, and innovation within a team<br>• Practices effective team behaviors	• Effectively delegates tasks to others and empowers others in their decisions<br>• Demonstrates effective team-building skills to achieve a shared goal	**Round 1**<br>Competency: 91%<br>Milestones: 90%<br>**Round 2**<br>Competency: 96%<br>Milestones: 91%

* There were 58 respondents in round 1 and 46 respondents in round 2 of the Delphi process. Delphi ratings are expressed as percentage that agree or strongly agree with the competency or milestone.

© 2024 Lee R et al. *JAMA Network Open.*

eTable 8. "Education" Final Focus Area Prefaculty Competencies and Milestones Proposed by an Expert Panel, With Ratings by Round, From a Modified Delphi Process, 2023*

Competency	Beginner	Intermediate	Advanced	Delphi Ratings
Recognizes education-related activities that align with personal values and professional interests	• Reflects on values and interests that drew them to serve as an educator<br>• Clarifies the relative importance of teaching as part of a future career	• Chooses specific education-related activities that best align with personal and professional interests	• Identifies and applies to an academic career track focused on education<br>• Reviews the promotion criteria for educators at institution of interest<br>• Prepares a teaching portfolio that aligns with personal values and professional interests	**Round 1**<br>Competency: 95%<br>Milestones: 88%<br>**Round 2**<br>Competency: 89%<br>Milestones: 83%
Engages in education-related experiences to build a foundation as an educator in academic medicine	• Lists various types of education-related activities<br>• Identifies an advisor, mentor, and/or sponsor focused on education<br>• Documents participation in educational activities	• Participates in a formalized educator training program (e.g., track, elective, certificate) during medical school, residency, or fellowship, if available<br>• Writes learning objectives using Bloom's taxonomy<br>• Incorporates adult learning principles into teaching activities<br>• Employs more than one teaching technique—lecture, small group facilitation, simulation, or other<br>• Seeks feedback on teaching skills	• Designs and uses a learner assessment tool<br>• Executes an evaluation of a learning activity or curriculum<br>• Demonstrates improvement in teaching based on feedback from learners	**Round 1**<br>Competency: 93%<br>Milestones: 88%<br>**Round 2**<br>Competency: 89%<br>Milestones: 87%
Transforms education-related activities into scholarship	• Identifies a mentor with experience in transforming education-related activities into scholarship<br>• Reflects on teaching activities to propose education research questions<br>• Reads and interprets an education research article<br>• Identifies gaps in the literature on an educational topic<br>• Differentiates frameworks to evolve educational work into scholarship<br>• Serves as a co-author on a poster or oral presentation to a group at their institution or on a local level	• Demonstrates fundamental educational skills in the design and implementation of an educational research project<br>• With supervision, designs and executes an education research project<br>• Serves as a lead author of a poster or oral presentation to a group on a regional level<br>• Serves as a coauthor on a peer-reviewed journal article or book chapter	• Serves as a lead author of a poster or oral presentation on the national or international level<br>• Serves as a lead author on a peer-reviewed journal article or book chapter	**Round 1**<br>Competency: 88%<br>Milestones: 90%<br>**Round 2**<br>Competency: 91%<br>Milestones: 87%

* There were 58 respondents in round 1 and 46 respondents in round 2 of the Delphi process. Delphi ratings are expressed as percentage that agree or strongly agree with the competency or milestone

eTable 9. "Community Engagement" Final Focus Area Prefaculty Competencies and Milestones Proposed by an Expert Panel, With Ratings by Round, From a Modified Delphi Process, 2023*

Competency	Beginner	Intermediate	Advanced	Delphi Ratings
Recognizes community-related activities that align with personal values and professional interests	● Reflects on community-based or advocacy activities and their alignment with personal and professional interests<br>● Clarifies the relative importance of community-based activities as part of a future career	● Chooses specific community-engaged activities that best align with personal and professional interests	● Prepares a community engagement/advocacy portfolio that aligns with personal values and professional interests<br>● Reviews promotion criteria for a faculty track focused on community engagement or advocacy	**Round 1**<br>Competency: 78%<br>Milestones: 84%<br>**Round 2**<br>Competency: 76%<br>Milestones: 79%
Engages in community service, community engagement, or advocacy activities as experiences leading to an academic career	● Defines the terms community service, community engagement, and service learning<br>● Describes the role of physician-advocate<br>● Participates in a community-based or advocacy project<br>● Identifies institutional and local advocates and policymakers to support advocacy activities<br>● Documents participation in community-based activities<br>● Describes how data can be used to identify community or stakeholder needs	● Identifies a "passion area" for community service or advocacy work<br>● Builds relationships with leaders or stakeholders in the community<br>● Uses data to plan a community service/ engagement/ advocacy activity<br>● Collects data to assess the impact of a community service/ advocacy project	● Recruits others or develops a team to engage in community service or advocacy activities<br>● Develops a continuity/ sustainability plan for community service or advocacy activities<br>● Identifies funding sources for community service and advocacy activities<br>● Identifies and applies to an academic career track focused on community engagement or advocacy<br>● Reviews the promotion criteria for community-based work at institution of interest	**Round 1**<br>Competency: 79%<br>Milestones: 86%<br>**Round 2**<br>Competency: 78%<br>Milestones: 83%
Transforms community-based and advocacy activities into scholarship to support an academic career	● Identifies a mentor with experience in transforming community-related activities into scholarship<br>● Reflects on community-based activities to propose a research question<br>● Reads and interprets a community-based research article<br>● Identifies gaps in the literature on a community-based topic<br>● Serves as a coauthor on a poster or oral presentation to a group at their institution or on local level	● Collects data to identify community needs and to assess impact of service or advocacy activities<br>● Utilizes frameworks to guide completion of community-based and advocacy scholarship<br>● Serves as a lead author of a poster or oral presentation to a group on regional level<br>● Serves as a coauthor on a peer-reviewed journal article or book chapter	● Serves as a lead author of a poster or oral presentation on the national or international level<br>● Serves as a lead author on a peer-reviewed journal article or book chapter	**Round 1**<br>Competency: 79%<br>Milestones: 83%<br>**Round 2**<br>Competency: 78%<br>Milestones: 83%

* There were 58 respondents in round 1 and 46 respondents in round 2 of the Delphi process. Delphi ratings are expressed as percentage that agree or strongly agree with the competency or milestone.

© 2024 Lee R et al. *JAMA Network Open.*

eTable 10. "Research" Final Focus Area Prefaculty Competencies and Milestones Proposed by an Expert Panel, With Ratings by Round, From a Modified Delphi Process, 2023*

Competency	Beginner	Intermediate	Advanced	Delphi Ratings
Recognizes research-related activities that align with personal values and professional interests (unless enrolled in combined MD/PhD or Medical Scientist Training Program)	• Reflects on values and interests that drew them to serve as a researcher<br>• Clarifies the relative importance of research as part of a future career<br>• Describes the process of creating a niche in scholarship and discovery	• Chooses specific research-related activities that best align with personal and professional interests<br>• Narrows research interests to a particular subject or domain	• Prepares a research portfolio that aligns with personal values and professional interests<br>• Identifies career options or faculty track that aligns with professional interest in research<br>• Reviews the promotion criteria for researchers at institution of interest	**Round 1**<br>Competency: 88%<br>Milestones: 86%<br>**Round 2**<br>Competency: 91%<br>Milestones: 87%
Engages in research to serve as experiences leading to an academic career	• Lists various types of research opportunities<br>• Identifies a research advisor, mentor, and/or sponsor<br>• Describes the elements of becoming a successful researcher or clinician scientist (grant writing, managing a lab or team, engaging with professional organizations, etc.)<br>• Lists research funding sources for trainees	• Participates in a formalized research program (e.g., track, elective, certificate) during medical school, residency, or fellowship if available<br>• Demonstrates fundamental research skills (e.g., question formulation, literature review, data collection methods, basic quantitative analysis) in the design, implementation, and/or evaluation of a project<br>• Participates in budget preparation for a research project<br>• Participates in and submits a grant proposal/funding request for research funding	• Creates a personal plan to pursue research<br>• Describes criteria for academic promotion for the chosen faculty track<br>• Negotiates a startup package that will support early career research<br>• Drafts funding approach for research project during first 5 years of academic practice	**Round 1**<br>Competency: 91%<br>Milestones: 90%<br>**Round 2**<br>Competency: 94%<br>Milestones: 94%
Transforms research activities into scholarly presentations and/or publications	• Identifies a mentor with experience in transforming research work into presentations or publications<br>• Identifies gaps in the literature on a topic of interest<br>• Serves as a coauthor on a poster or oral presentation to a group at their institution or on local level	• Serves as a lead author of a poster or oral presentation to a group on regional level<br>• Serves as a coauthor on a peer-reviewed journal article or book chapter	• Serves as a lead author of a poster or oral presentation on the national or international level<br>• Serves as a lead author on a peer-reviewed journal article or book chapter	**Round 1**<br>Competency: 93%<br>Milestones: 90%<br>**Round 2**<br>Competency: 98%<br>Milestones: 91%

* There were 58 respondents in round 1 and 46 respondents in round 2 of the Delphi process. Delphi ratings are expressed as percentage that agree or strongly agree with the competency or milestone.

© 2024 Lee R et al. *JAMA Network Open.*

eTable 11. "Clinical Medicine" Final Focus Area Prefaculty Competencies and Milestones Proposed by an Expert Panel, With Ratings by Round, From a Modified Delphi Process, 2023*

Competency	Beginner	Intermediate	Advanced	Delphi Ratings
Recognizes clinical medicine activities that align with personal values and professional interests	● Reflects on values and interests that drew them to serve as a clinician<br>● Clarifies the relative importance of clinical medicine as part of a future career	● Chooses specific clinical medicine–related activities that best align with personal and professional interests<br>● Narrows clinical medicine interests to a particular subject or domain	● Prepares a clinical medicine portfolio that aligns with personal values and professional interests<br>● Identifies career options or faculty track that aligns with professional interest in clinical medicine<br>● Reviews the promotion criteria for clinical faculty at institution of interest	**Round 1**<br>Competency: 90%<br>Milestones: 88%<br>**Round 2**<br>Competency: 91%<br>Milestones: 94%
Engages in clinical medicine to serve as experiences leading to an academic career	● Lists various types of clinical medicine opportunities<br>● Is able to differentiate clinical care team members based on their training or expertise<br>● Identifies a clinical medicine advisor, mentor, and/or sponsor<br>● Describes elements of successful clinical medicine faculty members (e.g., patient care, participation on patient care committees, patient safety, becoming an engaged member of the clinical care team, etc.)<br>● Describes how clinical care is funded (e.g., base and incentive, private and public insurance, etc.)<br>● Recognizes decision-makers that influence patient care<br>● Describes clinical priorities of the health system	● Participates in a formalized clinical medicine faculty program (e.g., track, elective, certificate) during medical school, residency, or fellowship<br>● Demonstrates appropriate billing and coding<br>● Develops practice management skills and supervision (e.g., review of performance, budget management, etc.)<br>● Engages in clinical leadership activities—quality assurance/ improvement, clinical pathway development, etc.<br>● Lists metrics for their clinical area	● Illustrates fundamental clinical medicine faculty skills (e.g., overseeing learners in clinical care, participating in health care system advisory groups and committees, etc.)<br>● Participates in budget preparation for a clinical medicine unit or department	**Round 1**<br>Competency: 93%<br>Milestones: 90%<br>**Round 2**<br>Competency: 89%<br>Milestones: 87%
Transforms clinical medicine–related activities into scholarship	● Describes the process of integrating clinical medicine into scholarship and discovery<br>● Identifies a mentor with experience in transforming clinical work into scholarship<br>● Identifies gaps in the literature on a topic of interest in clinical medicine	● Serves as a lead author of a poster or oral presentation to a group on a regional level (e.g., case presentation, review article, etc.)<br>● Serves as a coauthor on a peer-reviewed journal article or book chapter	● Serves as a lead author of a poster or oral presentation on the national or international level (e.g., presenting on alternative delivery schema)<br>● Serves as a lead author on a peer-reviewed journal article or book chapter	**Round 1**<br>Competency: 83%<br>Milestones: 86%<br>**Round 2**<br>Competency: 85%<br>Milestones: 87%

© 2024 Lee R et al. *JAMA Network Open.*

Competency	Beginner	Intermediate	Advanced	Delphi Ratings
	● Differentiates frameworks to evolve clinical medicine work into scholarship<br>● Serves as a coauthor on a poster or oral presentation to a group at their institution or on local level (e.g., case presentation, review article, etc.)			

* There were 58 respondents in round 1 and 46 respondents in round 2 of the Delphi process. Delphi ratings are expressed as percentage that agree or strongly agree with the competency or milestone.

© 2024 Lee R et al. *JAMA Network Open.*

eAppendix 1. BNGAP Publications

Zhang L, Lee E, Kenworthy C, et al. Southeast and East Asian American Medical Students' Perceptions of Careers in Academic Medicine. Journal of Career Development. 2019;46(3):235-250.

Sanchez NF, Callahan E, Brewster C, Poll N, Sánchez JP. The Future LGBT Health Professionals: Perspectives on Careers and Personal Mentorship. LGBT Health. 2018;5(3);212-220.

Sanchez N, Poll-Hunter N, Spencer D, et al. Attracting Diverse Talent to Academia: Perspectives of Medical Students and Residents. Journal of Career Development. 2018;45(5):440-457.

Sánchez JP, Poll-Hunter N, Stern N, Garcia AN, Brewster C. Balancing Two Cultures: American Indian/Alaska Native Medical Students' Perceptions of Academic Medicine Careers. J Community Health. 2016;41(4);871-80.

Sánchez N, Rankin S, Callahan E, et al. LGBT Trainee and Health Professional Perspectives on Academic Careers – Facilitators and Challenges. LGBT Health. 2015;2(4):346-56.

Yehia B, Cronholm P, Wilson N, et al. Mentorship and Pursuit of Academic Medicine.   Careers: A Mixed Methods Study of Residents from Diverse Backgrounds. BMC Medical Education. 2014;14(26). https://doi.org/10.1186/1472-6920-14-26

Castillo ND, Piserchio JP, Brewster C, Martinez D, Sánchez JP. Office of Diversity, Equity, and Inclusion: Engagement and Leadership Opportunities for Trainees. MedEdPORTAL. 2022;18:11282. https://doi.org/10.15766/mep_2374-8265.11282

Díaz DHS, Kothari P, Williams RL, Lee R, Mancias P, Davis JA, Sánchez JP. Office of Medical Education: Opportunities for Trainees to Engage and Lead in Curricular Innovation and Reform. MedEdPORTAL. 2021;17:11112. https://doi.org/10.15766/mep_2374-8265.11112

Nakae S, Haywood Y, Love LJ, Kothari P, Saldaña F, Sánchez JP. Office of Student Affairs: Engagement and Leadership Opportunities for Medical Students, Residents, and Fellows. MedEdPORTAL. 2021;17:11093. https://doi.org/10.15766/mep_2374-8265.11093

Lucas R, Kothari P, Adams C, Jones L, Williams VN, Sánchez JP. We are All Leaders: Introducing Self-Leadership Concepts Through the Lens of Improving Diversity in the Health Care Workforce. MedEdPORTAL. 2020;16:11011. https://doi.org/10.15766/mep_2374-8265.11011

Nakae S, Kothari P, Johnson K, Figueroa E, Sánchez JP. Office of Admissions: Engagement and Leadership Opportunities for Trainees. MedEdPORTAL. 2020;16:11018. https://doi.org/10.15766/mep_2374-8265.11018

Gilbert J, Kothari P, Sanchez N, Spencer DJ, Soto-Greene M, Sánchez JP. Is Academic Medicine a Financially Viable Career? Exploring Financial Considerations and Resources. MedEdPORTAL. 2020;16:10958. https://doi.org/10.15766/mep_2374-8265.10958

© 2024 Lee R et al. *JAMA Network Open.*

Nakae S, Parrish WP, Sánchez JP. Office of Admissions: Engagement and Leadership Opportunities for Medical Students. Acad Med. 2022 Feb 1;97(3):471.

Sola O, Kothari P, Mason HRC, Onumah CM, Sánchez JP. The Crossroads of Health Policy and Academic Medicine: An Early Introduction to Health Policy Skills to Facilitate Change. MedEdPORTAL. 2019;15:10827. https://doi.org/10.15766/mep_2374-8265.10827

Dickerman J, Sánchez JP, Portela-Martinez M, Roldan E. Leadership and Academic Medicine: Preparing Medical Students and Residents to Be Effective Leaders for the 21st Century. MedEdPORTAL. 2018;14:10677. https://doi.org/10.15766/mep_2374-8265.10677

Paredes Molina CS, Spencer DJ, Morcuende M, Soto-Greene M, Culbreath K, Corsino L, Sánchez JP. An Introduction to Research Work, Scholarship, and Paving a Way to a Career in Academic Medicine. MedEdPORTAL. 2018;14:10686. https://doi.org/10.15766/mep_2374-8265.10686

Guilliames C, Sule H, Perez H, Hubbi B, Sánchez JP. Providing Trainees With an Introduction and Decision-Making Framework for Pursuing an Academic Residency Position. MedEdPORTAL. 2018;14:10667. https://doi.org/10.15766/mep_2374-8265.10667

Nakae S, Soto-Greene M, Williams R, Guzman D, Sánchez JP. Helping Trainees Develop Scholarship in Academic Medicine From Community Service. MedEdPORTAL. 2017;13:10659. https://doi.org/10.15766/mep_2374-8265.10659

Callahan EJ, Banks M, Medina J, Disbrow K, Soto-Greene M, Sánchez JP. Providing Diverse Trainees an Early and Transparent Introduction to Academic Appointment and Promotion Processes. MedEdPORTAL. 2017;13:10661. https://doi.org/10.15766/mep_2374-8265.10661

Fernandez CR, Lucas R, Soto-Greene M, Sánchez JP. Introducing Trainees to Academic Medicine Career Roles and Responsibilities. MedEdPORTAL. 2017;13:10653. https://doi.org/10.15766/mep_2374-8265.10653

Williams R, Holaday L, Lamba S, Soto-Greene M, Sánchez JP. Introducing Trainees to Medical Education Activities and Opportunities for Educational Scholarship. MedEdPORTAL. 2017;13:10554. https://doi.org/10.15766/mep_2374-8265.10554

Sánchez JP, Brutus NN (Editors). Health Professions and Academia. Cham, Switzerland. Springer; 2022.

Sánchez, JP (Editor). Succeeding in Academic Medicine: A Roadmap for Diverse Medical Students and Residents. Cham, Switzerland. Springer; 2020.

© 2024 Lee R et al. *JAMA Network Open.*
