# Supplementary material for: Designing Prefaculty Competencies for Diverse Learners Through a Modified Delphi Process
Source: JAMA Netw Open. 2024 Jul 26;7(7):e2424003. doi: 10.1001/jamanetworkopen.2024.24003 (PMC11282442; doi:10.1001/jamanetworkopen.2024.24003)
Supplement: Supplement 2. — Data Sharing Statement [file jamanetwopen-e2424003-s002.pdf]

Data Sharing Statement

Lee. Designing Prefaculty Competencies for Diverse Learners Through a Modified Delphi Process. *JAMA Netw Open*. Published July 26, 2024.
doi:10.1001/jamanetworkopen.2024.24003

Data
Data available: No

Additional Information
Explanation for why data not available: Our expert panel respondents participated with the understanding that their responses would remain confidential.
